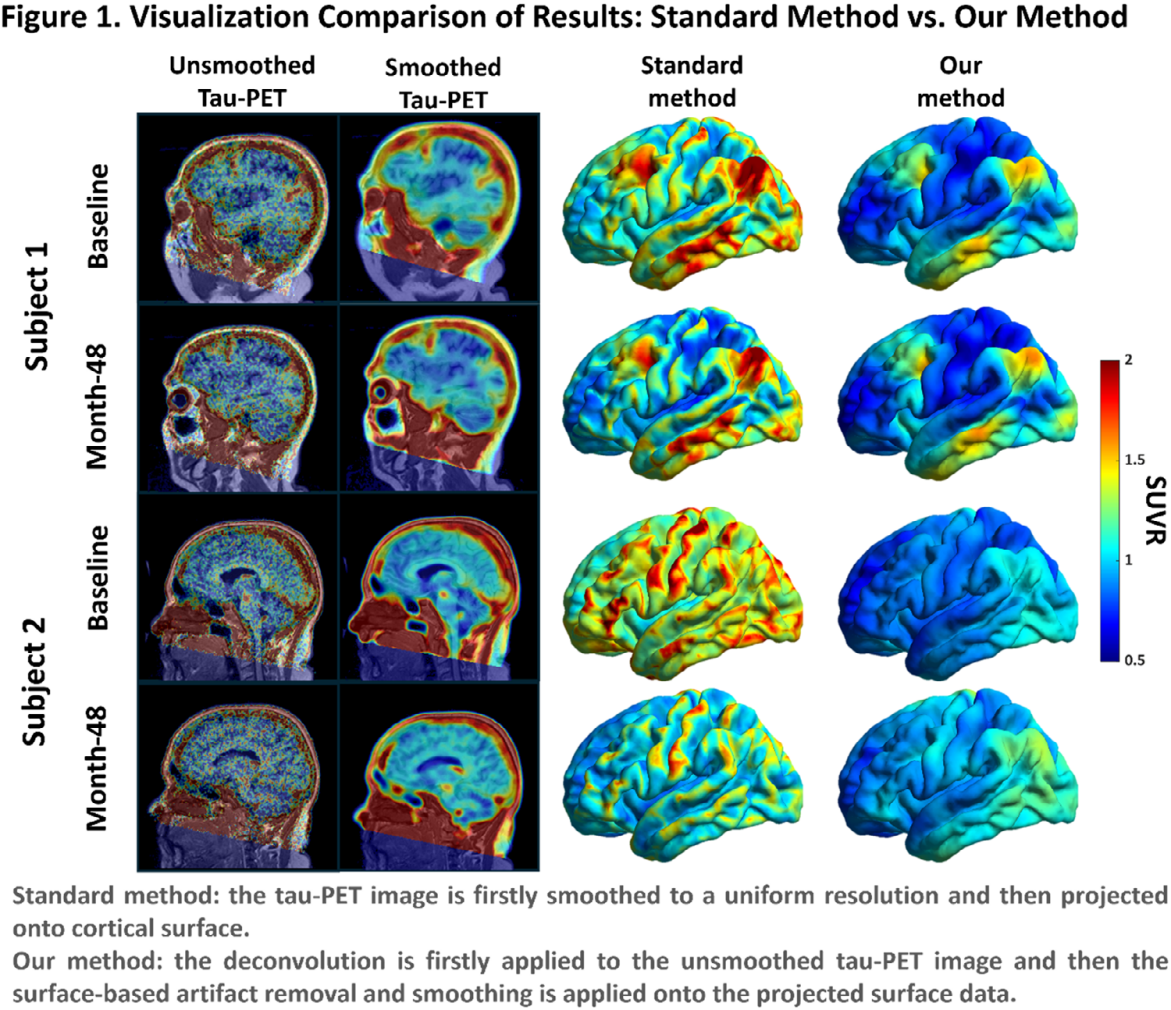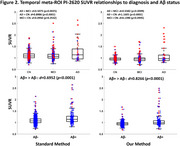# Voxel‐wise deconvolution and surface shape analysis for the correction of meningeal off‐target bindings in tau PET

**DOI:** 10.1002/alz70856_103659

**Published:** 2025-12-26

**Authors:** Jiaxin Yue, Jianwei Zhang, Yonggang Shi

**Affiliations:** ^1^ Stevens Neuroimaging and Informatics Institute, Keck School of Medicine, University of Southern California, Los Angeles, CA, USA; ^2^ University of Southern California, Los Angeles, CA, USA

## Abstract

**Background:**

Increasingly popular second‐generation tau PET tracers such as PI‐2620 and MK‐6240 often exhibits off‐target bindings in the meningeal areas. These artifacts lead to abnormally high uptake values on the cortex and complicate the interpretation of tau‐PET scans. To address this issue, we proposed a novel computational framework to effectively remove meningeal off‐target bindings and enhance the reliability of tau PET quantification on cortical regions.

**Method:**

First, we develop a voxel‐wise deconvolution to recover the original tau PET signal in the brain region to reduce spill‐in from neighboring meningeal regions caused by partial volume effects. Second, to further eliminate residual artifacts that persist after projecting the tau PET image onto cortical surfaces, we introduce a surface‐based artifact detection and removal method. Vertices that were spatially proximate to meningeal artifacts and displayed abnormal signal intensities were identified, and the values of these labeled vertices were reconstructed based on their connected neighbors on surface. Finally, to ensure consistent spatial smoothness and reduce noise, Laplacian smoothing was applied to the reconstructed surface data.

This framework was evaluated using the Health and Aging Brain Study: Health Disparities (HABS‐HD) dataset which employs PI‐2620 tau PET tracer. 758 subjects were used in the experiments, comprising 513 CN, 201 MCI and 44 AD subjects at baseline. Additionally, 216 subjects have 432 longitudinal scans, with an average interval of 24 months.

**Result:**

Our framework effectively removed spill‐in meningeal artifacts while preserving tau pathology on cortical surfaces (Figure 1). The authenticity of the pathology was further validated by improved longitudinal pattern consistency and the expected local monotonic increase of tau deposition over time. Besides, the average SUVRs at temporal meta‐ROI exhibit stronger associations with diagnosis (CN/ MCI/ AD) and Aβ status (Figure 2), as indicated by higher Cohen's *d* and lower *p*‐value. These findings suggest an enhanced diagnostic reliability of the processed tau PET data.

**Conclusion:**

Our proposed preprocessing framework effectively removes meningeal artifacts and enhances the diagnostic power of tau PET data on cortical surfaces. The improved data quality facilitates a better interpretation of tau pathology on the cortical surface.

**Funding Sources**: RF1AG077578, RF1AG064584, R01EB022744, U19AG078109, P30AG066530.